# Changes in entire acute bronchiolitis seasons before, during, and after the COVID-19 pandemic in Spain

**DOI:** 10.1016/j.infpip.2024.100399

**Published:** 2024-09-23

**Authors:** Juan Manuel Rius-Peris, María del Carmen Vicent Castelló, Marta Pareja León, Sara Pons Morales, Ana Amat Madramany, Jorge Pantoja-Martínez, Raquel Gil Piquer, Nuria Roda Martínez, Alicia Coret Sinisterra, Francisca Castillo Ochando, Francisco Javier Caballero Mora, María Teresa Moya Díaz-Pintado, ECEALHBA Project Research Group, J.M. Rius-Peris, A.I. Maraña Pérez, A. Valiente Armero, L. Guardia Nieto, J. Torrecilla Cañas, E.M. Cueto Calvo, C. Marcilla Vázquez, M. Pareja León, N. Roda Martínez, N. Molini Menchón, E. Felipe Almira, A. Párraga Cifuentes, J.M. Sequí-Canet, J.M. Olmos García, A. Martínez Bayo, P. Escrivá Tomás, S. Povo Martín, J. Pantoja-Martínez, F.J. Caballero Mora, L. García Maset, A. Coret Sinisterra, F. Castillo Ochando, M.J. Garrido Sánchez, A. Edo Tena, L. Vázquez Álvarez, L. Rabasco Álvarez, M.T. Moya Díaz-Pintado, I. Cardete Pascual, S. García Candel, A. Amat Madramany, M. Moreno López, S. Pons Morales, M.C. Vicent Castelló, A.M. Rivera Figueiras, A. Herrero Galiana, J. González de Dios, A. Domingo Pla, R. Gil Piquer

**Affiliations:** a Department of Paediatrics, Hospital Universitario Virgen de la Luz, Cuenca, Spain; b Department of Paediatrics, Hospital General Universitario de Alicante, Alicante, Spain; c Department of Paediatrics, Hospital General Universitario de Albacete, Albacete, Spain; d Department of Paediatrics, Hospital Universitario Doctor Peset, Valencia, Spain; e Department of Paediatrics, Hospital Universitario de La Ribera, Alzira, Valencia, Spain; f Department of Paediatrics, Hospital Universitario de La Plana, Villarreal, Castellón, Spain; g Department of Paediatrics, Hospital Lluís Alcanyís, Xátiva, Valencia, Spain; h Department of Paediatrics, Hospital de Sagunto, Sagunto, Valencia, Spain; i Department of Paediatrics, Hospital de Villarrobledo, Villarrobledo, Albacete, Spain; j Department of Paediatrics, Hospital Santa Bárbara, Puertollano, Ciudad Real, Spain; k Department of Paediatrics, Hospital Virgen de Altagracia, Manzanares, Ciudad Real, Spain

**Keywords:** Acute bronchiolitis, Infant, COVID-19, Epidemic season, RSV

## Abstract

**Background:**

Paediatric acute bronchiolitis normally occurs from October to April in the temperate northern hemisphere, peaking in January. Nonpharmacological measures to control the spread of COVID-19 resulted in a decrease in the number of cases of bronchiolitis during the 2020–21 season. The discontinuation of these measures created an uncertain scenario.

**Aim:**

To describe the epidemiological evolution of acute bronchiolitis seasons and the changes in the demographics of the affected population before, during, and after the implementation of nonpharmacological interventions for COVID-19 in Spain.

**Methods:**

This was a multicentre and descriptive study. A total of 6,334 infants aged up to 12 months who were diagnosed with acute bronchiolitis were recruited from sixteen Spanish hospitals. We collected data from participants from September 1st, 2021, through August 31st, 2023, as part of the ECEALHBA research project. The study periods were before (P1), during (P2), and after (P3) the COVID-19 pandemic.

**Findings:**

In P2 and after the discontinuation of nonpharmacological interventions, an unexpected increase in the number of acute bronchiolitis cases was observed from June–August 2021, resulting in an out-of-season peak. A subsequent peak was observed in November 2021, earlier than expected for the 2021-22 season. In the 2022-23 season, admissions followed a historical trend, with a greater number of cases than in the two previous seasons. Statistically significant differences in the length of stay (p<0.001), number of RSV infections (p=0.021), and number of paediatric intensive care unit admissions (p<0.001) were observed among the periods.

**Conclusions:**

Two out-of-season peaks in the number of acute bronchiolitis cases were observed in 2020–2021 and 2021–2022. However, following the relaxation of nonpharmacological intervention measures, the peak observed in 2022–2023, although occurring 2–6 weeks earlier, was more similar to the peaks observed in the prepandemic seasons. Additionally, increased case severity was observed during these periods.

## Introduction

Acute bronchiolitis (AB) is a respiratory infectious disease that mainly affects the peripheral bronchioles. It typically causes low-grade fever, nasal congestion, and rhinorrhoea, along with lower respiratory tract symptoms [[1], [2], [3]]. The continuous and rapid variation in clinical manifestations is a hallmark that can confound evaluation, which often requires several examinations over an observation period [2].

AB is the main cause of hospitalization in infants under two years of age. However, most children admitted during epidemic seasons are typically under 12 months of age, with a peak incidence usually occurring between 3 and 6 months of age [3,4]. Although several respiratory viruses can be responsible, respiratory syncytial virus (RSV) is the most common cause [5,6], accounting for more than half of the cases (56.4%) in Spain [7]. AB is a seasonal infection that usually extends from late October through April in the temperate northern hemisphere, with a peak in January-February [8].

At the end of 2019, the first cases of COVID-19 caused by SARS-CoV-2 were reported in Wuhan, China. Within a few months, the disease rapidly spread worldwide [9]. While some patients had mild or no symptoms, others experienced various symptoms of different degrees of severity [[10], [11], [12]]. In response to the increasing number of cases and deaths, in March 2020, the World Health Organization (WHO) declared COVID-19 a global pandemic [13]. Amidst this state of emergency and uncertainty, health care professionals and authorities anticipated the possibility of healthcare system collapse due in part to the potential co-occurrence of SARS-CoV-2 with other seasonal viruses, such as RSV or influenza [14].

The outbreak of the COVID-19 pandemic prompted the implementation of nonpharmacological public health interventions, including hand hygiene, masking, physical distancing, and travel restrictions, aimed at controlling the spread of the virus [15]. These measures likely contributed to a marked decrease in the number of cases of paediatric infectious diseases and admissions worldwide [[15], [16], [17], [18], [19], [20]], as well as changes in routine paediatric clinical practice [21,22]. In particular, during the COVID-19 pandemic, there was a marked decrease in the number of reported AB cases. Several European studies have shown noticeable deviations in the annual routine seasonality of bronchiolitis in settings where nonpharmacological measures were implemented [[23], [24], [25], [26], [27], [28]]. Reductions, varying between 80 and 95%, in the number of admissions were reported in European countries [26,27]. In Spain, the ECEALHBA (Spanish Collaborative Study for the Care of Hospitalized Infants with Acute Bronchiolitis) project was launched to describe the impact of the COVID-19 pandemic on AB cases, particularly during the 2020–21 epidemic season [28]. This multicentre study compared five epidemic seasons before the COVID-19 pandemic outbreak (from 2015 to 2020) to the 2020–2021 epidemic season, reporting a notable decrease in the number of bronchiolitis cases that could be related to the implementation of nonpharmacological public health interventions [28]. However, the effects of the relaxation of these measures on AB cases in the following seasons (2021–2022 and 2022–2023) were uncertain.

Therefore, our study aimed to describe the evolution of AB epidemic seasons and compare the clinical and demographic characteristics of admitted patients before, during and after the health emergency caused by the COVID-19 pandemic and to determine whether a possible relationship with the implementation of nonpharmacological measures exists.

## Methods

### Study design

This multicentre, ambispective, and descriptive study included infants aged ≤12 months with a diagnosis of AB. Sixteen Spanish hospitals participated in the data collection as part of the ECEALHBA research project. Prospective data collection was conducted between September 1^st^, 2021, and August 31^st^, 2023, to complement previously gathered data [28]. The variables included in the previous research and collected for the current study were the number of patients admitted to the hospital due to AB, date of birth, date of admission, date of discharge, sex, age, nationality, length of stay (LOS), RSV test result, and the need for admission to the paediatric intensive care unit (PICU). The changes in the number of AB cases over eight epidemic seasons (2015–16, 2016–17, 2017–18, 2018–19, 2019–20, 2020–21, 2021–22 and 2022–23) were analysed. These epidemic seasons spanned from September 1^st^ through August 31^st^ of the following year.

Three study periods were defined based on two key dates: 1) the declaration of the State of Alarm after the COVID-19 pandemic outbreak, which led to a national lockdown on 14^th^ March 2020 [29]; and 2) the end of 2021–22 RSV epidemic season (31^st^ August 2022), characterized by being the last season in which the seasonality and the number of RSV cases differed from prepandemic levels and by the relaxation of restrictions. These three periods were denoted as the *prepandemic* (P1, from 1^st^ September 2015 to 14^th^ March 2020), *pandemic* (P2, from 15^th^ March 2020 to 31^st^ August 2022), and *postpandemic* (P3, from 1^st^ September 2022 to 31^st^ August 2023) periods.

### Inclusion and exclusion criteria

Eligible infants were ≤12 months of age, required hospital admission, were diagnosed with AB, and were admitted to any of the hospitals participating in the study. AB was defined as the first presentation of a viral respiratory tract infection with respiratory distress. At the time of admission, the infants had not visited any health care provider (neither a GP nor a hospital physician) for these symptoms more than 1 month prior to the index visit [30]. The collected and analysed variables were the same as those described in a previous study [28]: the number of patients admitted for AB, admission and discharge dates, LOS in the hospital, diagnosis of RSV infection, admission to the PICU, and patient sociodemographic characteristics (birth date, sex, age, and nationality).

### Ethical considerations

The parents or guardians of all patients signed written informed consent forms for their inclusion. The study was approved by the Research Ethics Committee of Integrated Management of Cuenca as the promoting and coordinating hospital, as well as by all other collaborating hospitals. All procedures for the collection, transfer, and analysis of the data were in accordance with the Declaration of Helsinki and the regulatory standards of Organic Law 3/2018 (5^th^ December) on Personal Data Protection and Digital Rights.

### Statistical analysis

Qualitative variables are presented as absolute and relative frequencies. The chi-square test and Fisher's exact test were used as appropriate for comparisons. Quantitative variables, such as age and LOS, are presented as medians and interquartile ranges (IQRs). Due to their nonnormal distribution, the data were compared across different periods using the nonparametric Kruskal‒Wallis test. Post hoc pairwise comparisons were conducted using the Mann‒Whitney test to identify specific group differences. To adjust for multiple comparisons, we applied the Bonferroni correction to the significance levels. Statistical significance was set at p<0.05, and all analyses were performed using the Statistical Package for the Social Sciences (SPSS) version 21.0.

## Results

**Figure 1:**
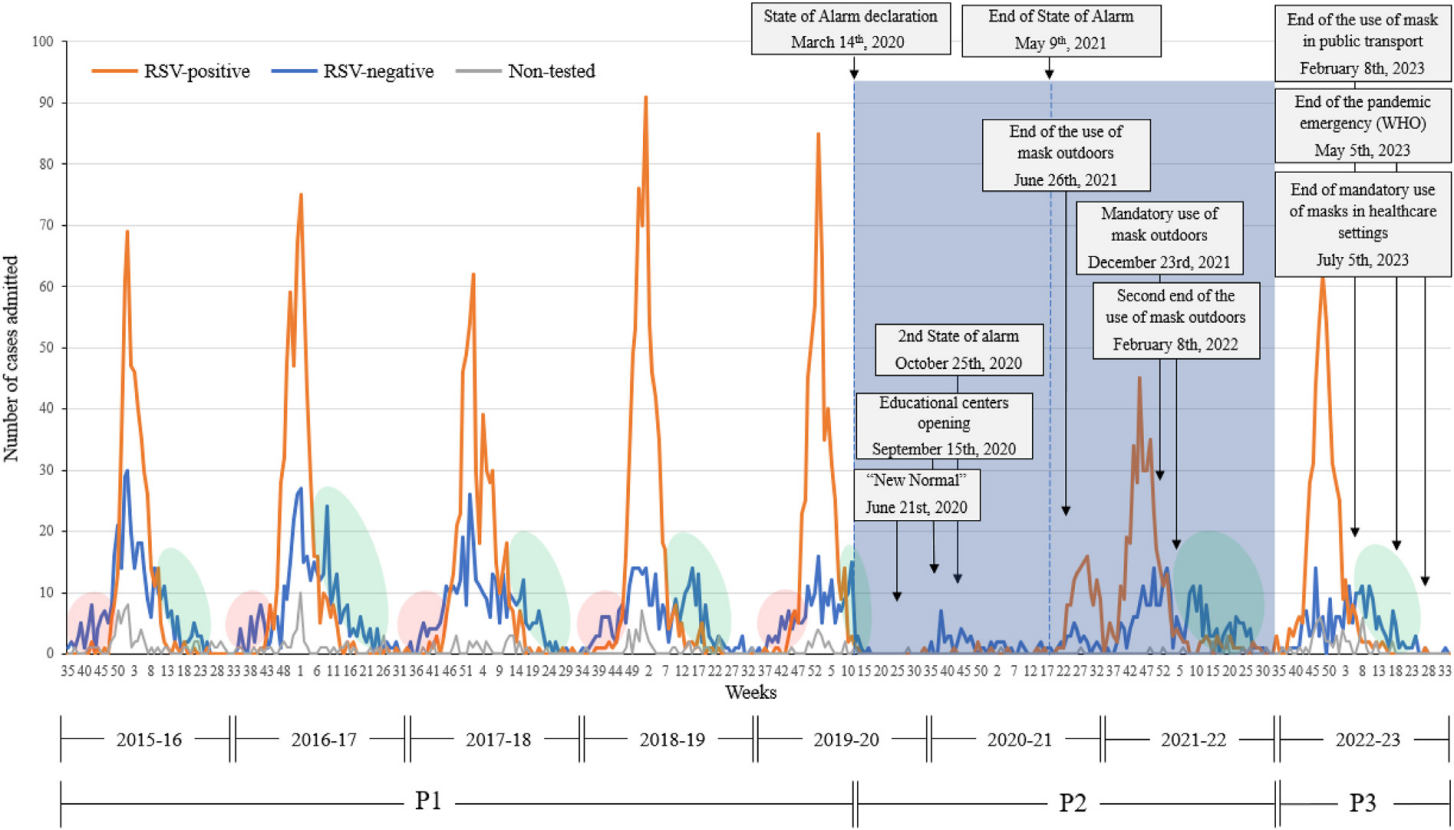
The distribution of acute bronchiolitis admissions during the study period was subdivided according to the aetiology of the disease. The red ellipses show the increase in the number of RSV-negative patients before the peak number of RSV-positive patients, and the green ellipses indicate the persistence of the number of RSV-negative patients after the decrease in the number of RSV-positive ones. P, Period; RSV, Respiratory Syncytial Virus; WHO, World Health Organization.

A total of 6,334 infants with AB were admitted to the participating hospitals during the eight epidemic seasons under study. Most infants were male (n=3,716; 58.7%), were aged three months or younger (median, 2.4; IQR, 1.4–4.5), were Spanish, and were hospitalized for 4–7 days (median, 4.05; IQR, 3.07–6.0) without being admitted to the PICU. Regarding the aetiology, 6,194 patients (97.8%) were tested for RSV, and 863 (13.6%) were tested for SARS-CoV-2. Of these patients, 3,864 (62.4%) and 20 (2.3%) tested positive, respectively (Table I). Most individuals were tested for RSV in all seasons. More than half of the patients tested positive for RSV (Table II). The data on the weekly distribution of cases during the epidemic seasons within the three considered periods are shown below (Figure 1, Table II).

**Table I:** Main characteristics of the patients included in the study

	Patients (n = 6,334)
Gender, n (%)	
Female	2,610 (41.3)
Male	3,716 (58.7)
Median age, months (IQR)	2.4 (1.4–4.5)
Age group distribution, n (%)	
Neonate	717 (11.3)
Infant ≤ 3 months	3,027 (47.8)
Infant > 3 months	2,589 (40.9)
Spanish nationality, n (%)	5,281 (83.8)
Length of stay, n (%)	
≤ 1 day	540 (8.5)
2–3 days	2,300 (36.3)
4–7 days	2,698 (42.6)
> 7 days	795 (12.6)
Median length of stay, days (IQR)	4.0 (3.0–6.0)
Admitted to the PICU, n (%)	580 (9.4)
Etiological study	
RSV positive, n (%)	3,864 (62.4)^a^
SARS-CoV-2 positive, n (%)	20 (2.3)^b^

IQR, interquartile range; PICU, pediatric intensive care unit; RSV, respiratory syncytial virus.

a RSV test performed in 6,194 patients (97.8% of the total sample). There were 140 missing cases.

b SARS-Cov-2 PCR test was performed in 863 infants (13.6% of the sample). The first case was diagnosed in October 2020. Among the SARS-Cov-2 positive cases, 9 were also RSV positive.

**Table II:** Start, end and peak of acute bronchiolitis seasons, number of admissions for each defined period, and aetiology of the disease

Periods			P1			P2		P3
Epidemic season	2015–16	2016–17	2017–18	2018–19	2019–20	2020–21	2021–22	2022–23
Start of season (week)^a^	47	45	43	44	47	22	38	41
End of season (week)^b^	22	16	22	24	12	34	27	18
Season peak (week)	1	52	1	1	2	28–29	45	48
Total duration (weeks)	21	21	28	33	18	13	19	28
Admissions (n)								
During the start week	5	10	5	4	10	2	4	8
During the end week	5	4	4	3	4	1	3	5
During the peak week	107	136	101	121	73	18	50	73
During epidemic season^c^	754	940	884	960	656	155	474	714
Total admissions per season^d^	857	1,013	928	995	766	221	673	881
Aetiology of the infection								
Tested for RSV infection, n (%)	791 (92.0)	969 (95.7)	900 (97.0)	955 (96.0)	744 (97.1)	219 (99.1)	660 (98.5)^e^	692 (93.0)^f^
RSV positive, n (%)	463 (58.5)	603 (62.2)	557 (61.9)	661 (69.2)	549 (73.8)	136 (62.1)	401 (61.0)	494 (71.4)
Tested for SARS-CoV-2, n (%)	-	-	-	-	-	201 (90.1)	662 (100.0)	-
SARS-CoV-2 positive, n (%)	-	-	-	-	-	5 (2.5)	15 (2.5)	-

RSV, respiratory syncytial virus.

a Denotes the week in which the number of hospital admissions began to increase significantly and gradually above the baseline level.

b Denotes the week in which the number of hospital admissions decreased and remained consistently below or at the level observed during the week immediately preceding the start of the epidemic season.

c All registered cases from the start week to the end week (epidemiological season).

d All registered cases during all the epidemiological year (from September 1^st^ to August 31^st^ of the following year).

e For the total admissions, there were three missing cases with no information regarding RSV testing.

f For the total admissions, there were 137 missing cases with no information regarding RSV testing.

In the prepandemic period, initial increases in the case count were observed in late October-early November (week 43–47), which ended around May–June (week 16–24), except in the 2019-20 season, which ended earlier (week 12, March), leaving a quantitatively lower number of cases than usually reported. The peak was reached by the end of December- beginning of January (week 52 or 1), and the seasons extended from 18–33 weeks. An increase in the number of RSV-negative patients before the peak number of RSV-positive patients was observed in all seasons in P1. The persistence of RSV-negative cases was observed after the number of RSV-positive patients decreased (Figure 1).

In P2, the reported number of admissions notably decreased. As observed in P2 (Figure 1), throughout the 2020-21 season, they remained practically nonexistent during the State of Alarm, with a slight rebound in the number of RSV-negative patients after the opening of educational centres. The last part of this season (between June and August 2021, weeks 22–34) showed an increase in the number of admissions, a 13-week season. Most of the patients tested positive for RSV by the time that the use of masks was no longer mandatory. Then, during the 2021–22 season, a second increase of cases was observed immediately after this relaxation, which extended from September 2021 to June 2022 (week 38–27). At approximately week 6, there was a decrease in the number of RSV-positive patients peak, and the second end of the use of face masks outdoors was announced (February 2022). In January–July 2022 (weeks 5–27), there was an increase in the number of RSV-negative patients. The number of COVID-19-positive patients in P2 was relatively low (Figure 1).

In P3, an increase in the number of admissions was observed in mid-October and lasted until early May (from weeks 41 to 18). The peak occurred in late November-early December (week 48), with values higher than those in the previous period, and the season lasted for 28 weeks. Furthermore, the number of cases throughout P3 (n=881) was greater than that throughout P2 (n=447 [average number of cases over two years]) and closer to the level described in the prepandemic seasons (n=912 [average of cases over five seasons in P1]; Table II). Most patients were RSV-positive and similar to those in P1. The number of RSV-negative patients persisted after the decrease in the number of RSV-positive patients, but there were no RSV-negative patients preceding the peak in RSV-positive patients (Figure 1).

A comparison of the clinical and demographic characteristics among the three study periods revealed a slight, statistically nonsignificant reduction in the median age of the patients when comparing P3 to P1 and P2 (p=0.193; Table III). In addition, differences in terms of the LOS among the three periods were noted (p< 0.001). A significantly greater median LOS was observed in P1 and P3 than in P2 (Table III). The number of PICU admissions increased from 8.0% during the entire prepandemic period (P1) to 10.1% in P2 and increased to 16.8% in P3 (p<0.001; Table III). However, in P2, there were significant differences between the 2020–21 and 2021-22 seasons (0.0% *vs*. 11.7%, p=0.02). Aetiological studies (RSV and SARS-CoV-2 tests) were conducted more frequently in P2 (p<0.081). Most admitted patients tested positive for RSV in the three periods, mostly in P3 (p=0.021; Table III).

**Table III:** Comparison of clinical and demographic characteristics of admitted infants with acute bronchiolitis among the three study periods

Variables	Pre-pandemic period (P1)	Pandemic period (P2)	Post-pandemic period (P3)	p-value
Gender, n (%)				
Female	1,903 (41.9)	377 (41.5)	336 (38.3)	0.070
Male	2,643 (58.1)	532 (58.5)	541 (61.7)	
Spanish nationality (n=5,281)	3,817 (84.6)	752 (82.7)	712 (81.1)	0.006^a^
Median age, months (IQR)	2.5 (1.4–4.5)	2.5 (1.4–4.9)	2.4 (1.4–4.4)	0.193
Median length of stay, days (IQR)	4.0 (3.0–6.0)	3.0 (2.0–5.0)	4.0 (2.0–6.0)	<0.001^b^ P1 *vs* P2; <0.001^c^ P1 *vs* P3; 0.059 P2 *vs* P3; 0.002^c^
Admitted to the PICU, n (%)	364 (8.0)	91 (10.1)	125 (16.8)	<0.001^a^
Etiological study done (n=5,930)	4,348 (95.6)	892 (98.5)	690 (92.7)	<0.081
RSV infection, n (%)				
Not tested	198 (4.4)	14 (1.5)	52 (7)	0.021^a^
Positive	2,830 (62.3)	540 (59.7)	494 (66.4)	
Negative	1,518 (33.4)	350 (38.7)	198 (26.6)	

IQR, interquartile range; PICU, paediatric intensive care unit; RSV, respiratory syncytial virus.

a Statistically significant according to the Chi-square test.

b Statistically significant according to the Kruskal-Wallis test.

c Statistically significant according to the post hoc pairwise comparisons using the Mann-Whitney.

## Discussion

We previously reported a marked reduction in the number of AB cases during the State of Alarm in Spain [28], which was in accordance with published data from other European countries [26,27]. This decline has been related to the implementation of nonpharmacological interventions that could have prevented the transmission of respiratory viruses, including SARS-CoV-2 [[31], [32], [33]]. However, there was uncertainty regarding the consequences of relaxing these measures on these seasonal diseases. In this study, we examined the epidemiology of AB after the relaxation of COVID-19 restrictions in Spain and compared it with that observed before and during the implementation of measures in response to the COVID-19 pandemic. This study adds information to our previous findings [28], offering a complete picture of the potential impact of nonpharmacological interventions on the number and distribution of AB cases and the changes after their relaxation.

In addition to the notable decrease in admissions during the State of Alarm, a slight rebound of the number of RSV-negative patients, mainly caused by rhinovirus infection, was reported, while the number of RSV-positive patients remained minimal [28]. This coincides with an apparent reduction in the severity of AB cases (shorter stays in the hospital and a lower number of PICU admissions between March 2020 and May 2021). The differing efficacy of nonpharmacological interventions against viruses [34] and viral interference [35,36] may have contributed to changes in aetiology and severity throughout this period.

An increase in the number of AB cases was recorded in Spain just after the end of the State of Alarm, possibly due to the discontinuation of most nonpharmacological interventions, even though it was an atypical period. There were two peaks, predominantly involving RSV-positive patients, although a slight increase in the number of RSV-negative patients was also observed shortly after, similar to that in the prepandemic season (Figure 1). Other Spanish hospitals independently reported similar data, noting an out-of-season increase in the number of admissions, with an unusual summer peak in 2021 [24,[37], [38], [39]]. However, these single-centre studies only covered up to March 2022, capturing only the initial short summer peak [24,37,38]. Only one study reported the second out-of-season peak in September 2021 [39].

Other European countries published data consistent with our results. An unexpected season of RSV activity was reported in England during the summer of 2021, when most epidemic indicators (i.e., RSV cases, hospital admissions, emergency and nonemergency visits, or general practitioner consultations) were lower than those observed in prepandemic seasons but higher than those observed in the 2020-21 season [40]. Similarly, in France, a delayed RSV infection season was reported during 2020–21 [41]. An Italian study also described a “post-COVID period” characterized by an early and short-term peak starting in September 2021 [42]. Notably, similar trends were also observed during the pandemic period for other seasonal respiratory viruses, such as influenza [43]. This atypical increase in the number of AB cases may have occurred for two possible reasons. The first out-of-season peak could correspond to the peak that was expected in the 2020-21 season but was delayed due to the implementation of nonpharmacological measures [24,41]. The second possible reason is the anticipation of the 2021–22 epidemic season following the relaxation of these measures [39]. Interestingly, these shifts seemed to be exclusive to cases of AB caused by RSV infection, while those with different aetiologies remained typical during the 2021–22 season.

For the 2022-23 season, the number and timing of RSV admissions closely resembled prepandemic data. This period had a slightly earlier peak than the prepandemic one but later than the previous season, accompanied by an increase in the total number of admissions due to AB as well as RSV-positive infants, indicating a return to the usual seasonality of RSV. Similarly, Vitucci *et al.* [44] compared the RSV trends among hospitalized infants between the 2022–2023 season and the last prepandemic season (2018–19) and reported an earlier onset, a nearly identical LOS, and a higher median age at admission*.* In the United States, a return to prepandemic seasonality was also observed during the 2022-23 season, along with an increase in the intensity of RSV circulation [45].

However, we observed a significant increase in the number of PICU admissions after the end of the State of Alarm, suggesting greater illness severity and possibly greater RSV virulence. Although similar LOS appear to mitigate the apparent increase in case severity, this may be attributed to measures adopted to prevent the spread of COVID-19, including recommendations to limit the LOS in health care facilities, particularly for vulnerable populations. In this context, Curatola *et al.* [42] reported increased virulence of RSV during the “post-COVID period” in Italy, with hospitalization rates increasing from ∼25% in the “pre-COVID-19” and “COVID-19” periods to ∼33% in the “post-COVID-19” period. The LOS and aetiology also varied significantly among cases reported in each period. Interestingly, three other studies conducted in Italy also documented an increase in the severity of AB cases during the 2021–22 and 2022-23 seasons compared to the prepandemic seasons [[46], [47], [48]]. Similarly, a single-centre Spanish study reported statistically significant differences in age, the PICU admission rate, hospital LOS, and the aetiology of bronchiolitis cases during the season with a delayed peak compared to previous seasons [38]. The apparent increase in the severity of AB cases was attributed to the unusual absence of cases during the 2020–21 season [40,42,49].

It seems unlikely that COVID-19 vaccination could have influenced the changes in bronchiolitis seasons. In fact, the literature and previous experience point to nonpharmacological interventions as potentially responsible for these changes. The establishment of nonpharmacological interventions could reduce the transmission of respiratory viruses causing AB. As a result, the newborn population may not have developed immunity to these viruses, creating an “immunity debt” and thereby increasing the risk for infection when measures were withdrawn [40,42,49]. In addition, during and after the State of Alarm, more aetiological studies were performed. Probably, this fact agreed with the need to identify any potential cases caused by SARS-CoV-2.

On May 5^th^, 2023, the WHO declared the end of the pandemic emergency. In Spain, the mandatory use of masks was revoked two months later, and the influence of the pandemic on infant admissions ceased, with AB timing returning to levels seen in prepandemic seasons. We can hypothesize that the incidence of AB will decrease in the 2023-24 season, given the approval of nirsevimab (a monoclonal antibody) for widespread immunization against RVS in neonates and infants during their first season of exposure to RSV. This study will serve as a valuable tool for comparing the preceding periods with the upcoming season characterized by this immunization.

Our study represents the first multicentre analysis of the effect of the relaxation of nonpharmacological COVID-19 control measures after the end of the State of Alarm on changes in AB seasons in Spain. To our knowledge, no previous research has presented complete data for a full epidemic year after the pandemic period (2022-23 season). Our data shed light on what has happened with AB after the relaxation of measures for the prevention of COVID-19 spread. With the COVID pandemic now declared over, the seasonality and number of RSV cases have returned closer to prepandemic levels. While this points to the potential role of nonpharmacological interventions in the control and prevention of seasonal viruses, future directions may involve studying the impact of pharmacological interventions, including nirsevimab, a monoclonal antibody recently approved for immunization against RSV-associated AB.

However, the present study is not exempt from limitations. First, given the variability and differences among Spanish regions in the type and duration of nonpharmacological measures implemented, it is difficult to isolate the individual effects of different interventions. Thus, direct causality cannot be confirmed, but a relationship between the relaxation of these control measures and the rebound of AB cases can be highlighted. Moreover, the study population included only patients who visited the participating hospitals, which could have led to bias towards the Eastern and Central Spanish populations. It would be interesting to complete these data by extending the study period and including data from other Spanish regions. It is also worth mentioning that in the 2022-23 season, the AB aetiology tests were highly heterogeneous, and a considerable number of patients were lost to RSV testing.

## Conclusions

Our data shed light on the effect of the relaxation of measures for the prevention of COVID-19 on AB seasons. With the COVID pandemic now declared over, the seasonality and number of RSV cases have returned closer to prepandemic levels. While this points to the potential role of nonpharmacological interventions in the control and prevention of seasonal viruses, future directions may involve studying the impact of pharmacological interventions, including nirsevimab, a monoclonal antibody recently approved for immunization against RSV-associated AB.

## Credit author statement

**Dr. Rius Peris:** Conceptualization, Methodology, Formal analysis, Investigation, Writing – Original Draft, Writing – Review & Editing, Supervision, Project administration. **Dr. Vicent Castelló:** Investigation, Writing – Review & Editing. **Dr. Pareja León:** Investigation, Writing – Review & Editing. **Dr. Pons Morales:** Investigation, Writing – Review & Editing. **Dr. Amat Madramany:** Investigation, Writing – Review & Editing. **Dr. Pantoja Martínez:** Investigation, Writing – Review & Editing. **Dr. Gil Piquer:** Investigation, Writing – Review & Editing. **Dr. Roda Martínez:** Investigation, Writing – Review & Editing. **Dr. Coret Sinisterra:** Investigation, Writing – Review & Editing. **Dr. Castillo Ochando:** Investigation, Writing – Review & Editing. **Dr. Caballero Mora:** Investigation, Writing – Review & Editing. **Dr. Moya Díaz-Pintado:** Investigation, Writing – Review & Editing.

## Conflict of interest statement

None.

## Funding

This work was supported by Sanofi. Sanofi has funded the medical writing and editorial support of this manuscript. The sponsor did not participate in study design; collection, analysis, and interpretation of data; writing of the report; or in the decision to submit the article for publication. The authors were not paid for writing the publication.

## Ethics approval

This study was approved by the Ethics Research Committee of Integrated Management of Cuenca (01122019).

## Appendix

ECEALHBA Project Research Group is formed by:

Rius-Peris JM; Maraña Pérez AI; Valiente Armero A; Guardia Nieto L; Torrecilla Cañas J; Cueto Calvo EM. Hospital Virgen de la Luz. Cuenca, Spain.

Marcilla Vázquez C; Pareja León M; Roda Martínez N. Hospital General de Albacete. Albacete, Spain.

Molini Menchón N. Hospital General de Castellón. Castellón, Spain.

Felipe Almira E; Párraga Cifuentes A; Sequí-Canet JM. Hospital Francisco Borja. Gandía, Spain.

Olmos García JM; Martínez Bayo A; Escrivá Tomás P. Hospital Virgen de los Lirios. Alcoy, Alicante, Spain.

Povo Martín S; Pantoja-Martínez J. Hospital La Plana. Villarreal, Castellón, Spain.

Caballero Mora FJ. Hospital Santa Bárbara. Puertollano, Ciudad Real, Spain.

García Maset L; Coret Sinisterra A. Hospital de Sagunto. Sagunto, Valencia, Spain.

Castillo Ochando F; Garrido Sánchez MJ. Hospital de Villarrobledo. Villarrobledo, Albacete, Spain.

Edo Tena A; Vázquez Álvarez L; Rabasco Álvarez L. Hospital de Vinaroz, Vinaroz, Castellón, Spain.

Moya Díaz-Pintado MT. Hospital Virgen de Altagracia. Manzanares, Ciudad Real, Spain.

Cardete Pascual I; García Candel S; Amat Madramany A. Hospital de La Ribera. Alzira, Valencia, Spain.

Moreno López M. Hospital de Tomelloso. Tomelloso, Ciudad Real, Spain.

Pons Morales S. Hospital Doctor Peset. Valencia, Spain.

Vicent Castelló MC; Rivera Figueiras AM; Herrero Galiana A; González de Dios J. Hospital General de Alicante, Alicante, Spain.

Domingo Pla A; Gil Piquer R. Hospital Luís Alcañiz. Játiva, Valencia, Spain.
